# Social evolution in micro-organisms and a Trojan horse approach to medical intervention strategies

**DOI:** 10.1098/rstb.2009.0055

**Published:** 2009-11-12

**Authors:** Sam P. Brown, Stuart A. West, Stephen P. Diggle, Ashleigh S. Griffin

**Affiliations:** 1 Department of Zoology, University of Oxford, South Parks Road, Oxford OX1 3PS, UK; 2 School of Molecular Medical Sciences, Centre for Biomolecular Sciences, University Park, University of Nottingham, Nottingham NG7 2RD, UK

**Keywords:** altruism, bacteriocins, cheat, cooperation, spite, virulence

## Abstract

Medical science is typically pitted against the evolutionary forces acting upon infective populations of bacteria. As an alternative strategy, we could exploit our growing understanding of population dynamics of social traits in bacteria to help treat bacterial disease. In particular, population dynamics of social traits could be exploited to introduce less virulent strains of bacteria, or medically beneficial alleles into infective populations. We discuss how bacterial strains adopting different social strategies can invade a population of cooperative wild-type, considering public good cheats, cheats carrying medically beneficial alleles (Trojan horses) and cheats carrying allelopathic traits (anti-competitor chemical bacteriocins or temperate bacteriophage viruses). We suggest that exploitation of the ability of cheats to invade cooperative, wild-type populations is a potential new strategy for treating bacterial disease.

## 1. Introduction

Bacteria and other micro-organisms exhibit a wide range of social behaviours. Technological advances made by microbiologists have overturned the long held assumption that micro-organisms live relatively independent, unicellular lives. Instead, it appears that individual cells can communicate and cooperate to perform activities such as dispersal, foraging, construction of biofilms, reproduction, chemical warfare and signalling (reviewed by Crespi 2001; Velicer 2003; Webb *et al*. 2003; Keller & Surette 2006; Kolter & Greenberg 2006; West *et al*. 2006; Diggle *et al*. 2007*a*; Foster 2007; Hense *et al*. 2007; West *et al*. 2007*a*; Williams *et al*. 2007; Brown & Buckling 2008; MacLean 2008; Popat *et al*. 2008). These social behaviours are comparable to the more familiar patterns of sociality in metazoans such as social insects and cooperative breeding vertebrates, discussed in the other articles in this volume. Furthermore, these social traits are critical to determining the damage caused by microbial parasites to their hosts (virulence) due to their importance for microbial growth.

**Figure 1.**
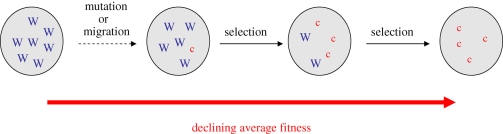
Natural selection favours selfish individuals who do not cooperate. Consider a population of wild-type cooperators (‘W’) in which an uncooperative, selfish cheater (‘c’) arises through mutation or migration. In a mixed population, the selfish cheater benefits from the cooperative behaviour of the cooperators, without paying the cost. Consequently, the selfish cheater has a higher fitness than the cooperators and spreads through the population, despite the fact that this leads to a decline in mean fitness (redrawn from Nowak 2006; West *et al*. 2007*b*). This figure illustrates the problem of cooperation—our main aim in this paper is to discuss how this problem can be exploited for medical intervention strategies.

However, evolutionary theory shows how cooperation is easily lost in competition against selfish strategies (Hamilton 1964; Trivers 1971). Consider a population of unconditional cooperators in which an uncooperative, relatively selfish cheater arises through mutation or migration. In the absence of any mechanism to punish non-cooperators, the cheater benefits from the cooperative behaviour of its social partners, without paying any cost. Consequently, genes for cheating have greater fitness than the genes for cooperation, and spread through the population, even though this will lead to a decline in population fitness (figure 1). A large body of theoretical and empirical work has examined the conditions under which cooperation can be favoured, via direct or indirect (kin selected) benefits (reviewed by Sachs *et al*. 2004; Lehmann & Keller 2006; West *et al*. 2007*b*), and how this may be applied to microbes (West *et al*. 2006).

Experimental studies using microbes show how cheats can invade populations of wild-type cooperators (Strassmann *et al*. 2000; Velicer *et al*. 2000; Greig & Travisano 2004; Griffin *et al*. 2004; Harrison *et al*. 2006; Diggle *et al*. 2007*b*; Ross-Gillespie *et al*. 2007; Sandoz *et al*. 2007; Kümmerli *et al*. 2009*a*,*b*; Rumbaugh *et al*. 2009). The most common way for microbes to cooperate with one another is by the release of exoproducts, such as proteases and toxins, that facilitate bacterial growth (West *et al*. 2007*a*). Exoproducts can benefit any individual in the local group and so can be considered as public goods, which can be exploited by cheats that do not produce the exoproduct. Possibly, the best studied exoproducts from a evolutionary perspective are iron-scavenging siderophore molecules in *Pseudomonas aeruginosa* (West & Buckling 2003). Mutants (cheats or free-riders) that do not produce siderophores are able to exploit those produced by others, and hence increase in frequency in mixed populations that contain both cooperators and cheats (Griffin *et al*. 2004; Harrison *et al*. 2006). Another layer of complexity is that the release of many exoproducts is regulated in a cell density-dependent manner via diffusible signal molecules by a process that has been termed quorum sensing (QS). Experimental studies in both laboratory cultures and mouse hosts have shown that QS-defective mutants, that do not signal or respond to signal, are able to benefit from public goods produced by others and increase in frequency, even in conditions where they would normally show only very limited growth (Diggle *et al*. 2007*b*; Sandoz *et al*. 2007; Rumbaugh *et al*. 2009).

**Figure 2.**
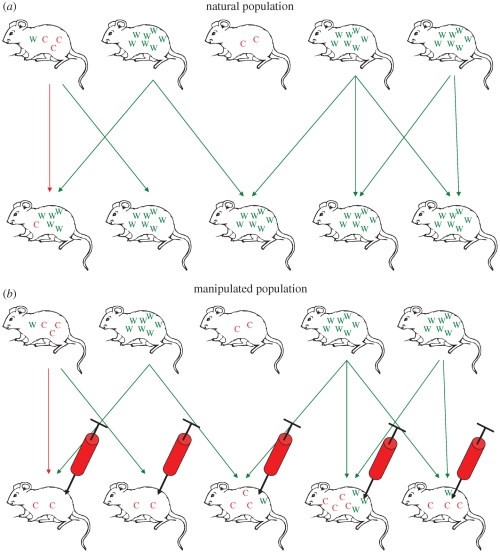
Manipulation of natural population dynamics of social behaviour to reduce virulence of bacterial infection. The top row of mice represents the initial population of hosts which are infected with either wild-type (W in green) or cheat (C in red) strains of bacteria. The arrows represent transmission to new hosts, represented by the lower row of mice. (*a*) Illustrates the fact that wild-type strains are more productive and better at transmission and colonization of new hosts. This promotes the maintenance of cooperative behaviour in the global population: even though cheats can outcompete wild-type strains within hosts, groups containing only wild-type will be more productive and more likely to spread to new hosts (this requires that wild-type and cheats tend to be in different hosts—high relatedness). (*b*) Illustrates how inoculation of hosts containing wild-type infections with cheater strains can counteract the natural population dynamics of cooperators and cheats.

Cooperative behaviours in bacteria are of particular interest because they are fundamental to the success and virulence of bacterial infections. Exoproducts are commonly referred to as ‘virulence factors’ because their production is associated with virulence, either through direct damage to the host, or through aiding bacterial growth (West *et al*. 2007*a*). Infections containing mutants that do not produce exoproducts are often characterized by lower virulence. For example, QS has been demonstrated to be important for virulence in several species of bacteria including *P. aeruginosa* (Rumbaugh *et al*. 1999, 2009), *Burkholderia pseudomallei* (Ulrich *et al*. 2004), *Vibrio cholerae* (Lin *et al*. 2007) and *Staphylococcus aureus* (Fleming *et al*. 2006). In nature, we do not see cheats dominate because infections that contain few cooperators will be less productive and relatively poor in transmission and initiating new colonies (Brown & Johnstone 2001; Diggle 2007*a*; Diggle *et al*. 2007*b*; Rumbaugh *et al*. 2009; see figure 2 and discussion). However, the population structure of pathogen populations that favours the evolution of cooperative, virulence traits (figure 2) presents an opportunity for the artificial introduction of social cheats, and consequent disruption of microbial-cooperative virulence traits (André & Godelle 2005).

**Figure 3.**
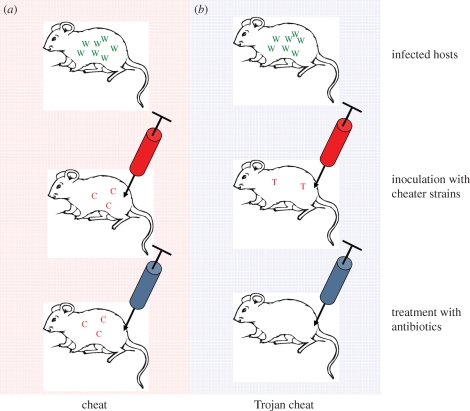
Introducing antibiotic resistance with a Trojan horse approach. (*a*) This panel shows a host infected with wild-type strains (W in green) inoculated with a strain of cheats (C in red) that are able to invade and outcompete the wild-type infection. The resulting population of bacteria is less virulent, less productive and more vulnerable to eradication by the host's immune system. However, this cheat strain (C) is still resistant to antibiotics. (*b*) This panel represents a host inoculated with a cheater strain (T in red) that has also been genetically engineered to have antibiotic sensitivity restored, allowing the infection to be eradicated by antibiotic treatment. We have called this kind of strain a Trojan horse cheat: host = Troy; cheat = wooden horse; antibiotic sensitive gene = Greeks. This representation shows the best possible outcome: in all likelihood, the cheats will not completely eradicate the wild-type cells and so, following antibiotic treatment, the resistant population could recover. Even if resistant strains survive, the infective population may be reduced to levels that can be eradicated by immune system of the host and there is theoretically no limit to the number of times antibiotic-sensitive strains could be inoculated.

We review the possibility of exploiting the ability of cheater strains to invade cooperative, wild-type populations in medical intervention strategies. Firstly, the introduction of an invasive cheat can lead to direct reduction in parasite virulence, as well as a reduced bacterial population size, that may make the infection more susceptible to other intervention strategies (Harrison *et al*. 2006; West *et al*. 2006; Diggle *et al*. 2007*b*; Kurzban & Egreth 2008; Rumbaugh *et al*. 2009). A second possibility is that cheats could act as ‘Trojan horses’, vehicles for the introduction of alleles such as sensitivity to antibiotics, into a population that was previously antibiotic-resistant (figure 3). Another possible use for the Trojan horse approach would be to introduce a lethal toxin under the control of an inducible promoter, which when activated, would eliminate both cooperators and cheats. We present a number of heuristic models to formally illustrate the possibilities as simply as possible.

## 2. Population dynamics of social traits

In this section, we show how social traits may be exploited as part of a medical intervention strategy. We first focus on cooperative social traits, such as the production of exoproducts (cooperative public goods). In §2*a* we show how a cheat that does not produce some exoproduct can invade a wild-type population, consisting of cooperative individuals who do produce the exoproduct. This can lead to a direct decrease in parasite virulence (due to loss of exoproduct production), as well as a smaller population size that may be more susceptible to other intervention strategies (e.g. antibiotic treatment). In §2*b* we extend this, by showing how such traits can also be used to hitch-hike useful traits (e.g. antibiotic vulnerabilities) into the population. In §2*c* we examine the complications that can occur when within-host populations are spatially structured, which will reduce the ability of non-cooperative cheats to spread.

We then consider how harmful, or spiteful, social traits may be exploited, such as anti-competitor chemicals, bacteriocins and temperate bacteriophage viruses. In §2*d*, we show that the addition of bacteriocin production to a cheat or Trojan horse lineage can favour the engineered strain's invasion, particularly in spatially structured host compartments. We then discuss how related forms of microbial spite involving the production of temperate phages can generate distinct invasion advantages to an engineered strain, in particular, allowing rapid invasion from rare into relatively unstructured foci of infection.

### (a) A cheat that does not produce exoproducts (public goods)

Possibly, the most common form of social behaviour in microbes is the production of exoproducts. Exoproducts are manufactured by an individual, but can then be used by the individual and its neighbours. For example, bacteria produce numerous factors that are released into the environment beyond the cell membrane, such as siderophores to scavenge iron, proteases to digest proteins and β-lactamases to inactivate antibiotics (see table 1 of West *et al*. 2007*a*). Exoproducts lead to the problem of cooperation because they are metabolically costly to the individual to produce but provide a benefit to all the individuals in the local group or population, as well as the individual that produced them, and hence can be thought of as public goods (West *et al*. 2006). Such exoproducts are often termed ‘virulence factors’, because their production is linked with damage to the host, either through some direct effect, or through facilitating parasite growth.

**Table 1.** A classification of social strategies by their conjectured strengths.

strategy	favoured by
cheat	unstructured populations, more highly diffusible exoproducts, more costly exoproducts, lower specificity in exoproduct uptake
Trojan horse cheat	as above, and with low cost or high benefit associated with Trojan horse trait
Trojan horse cooperator	structured populations, less diffusible exoproducts, less costly exoproducts, higher specificity in exoproduct uptake
bacteriocin cheat	structured populations (if low cheat advantage), more diffusible bacteriocins, higher specificity in bacteriocin immunity, low cost or high benefit associated with any Trojan horse trait
lysogen cheat	unstructured populations, durable phage propagules, low cost or high benefit associated with any Trojan horse trait

We begin with a simple ecological model of within-host competition between a cooperative, resident wild-type lineage that produces a certain exoproduct, and an invading cheat lineage that does not produce the exoproduct, but can profit equally from its presence within the host. Such cheats can be genetically engineered, artificially selected for and are found in natural populations. For illustration, we assume no within-host population structuring—this assumption is relaxed below. The within-host cell densities of the wild-type and cheat are *W* and *C*, respectively, and we describe their change in time via the following ordinary differential equations:

2.1
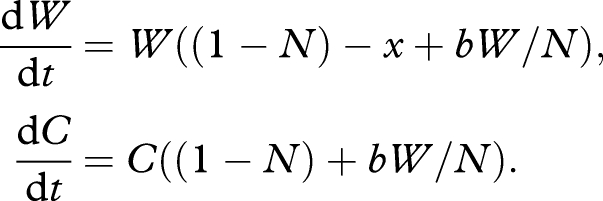


**Figure 4.**
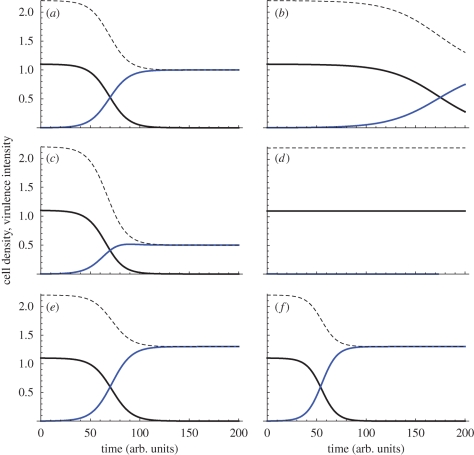
Invasion of cheats and Trojan horses into an established resident infection and consequent virulence (model 4). Black lines, wild-type (*W*). Blue lines, cheats or Trojan horses (*C* or *T*). Dotted lines, virulence (a weighted sum of wild-type and cheat density). (*a*,*c*,*e*) No within-host spatial structure (*r* = 0). (*b*,*d*,*f*) Limited within-host spatial structure (*r* = 0.3). (*a*,*b*) Cheats versus wild-type (model 4 with *a* = *q* = 0); virulence is a weighted combination of the two lineage densities, here virulence equals 2*W* + *C*. (*c*,*d*) Trojan horse cheats versus wild-type (model 4 with *a* = 0.5, *q* = 0.01), virulence equals 2*W* + *T*. (*e*,*f*) Trojan horse cooperators versus wild-type (model 4 with *a* = −0.3, *q* = 0.01), virulence equals 2*W*+*T*. Other parameters: *x* = 0.1, *b* = 0.2. Initial densities: *W*(0) = 1.1, *C*(0) = 0.001, *T*(0) = 0.001.

Here, *x* is the cost of exoproduct production, *b* the benefit (weighted by frequency of wild-type, *W/N*) and *N* total population within the host (*N* = *W* + *C*). In the absence of any exoproduct production (*x* = *b* = 0), we have a simple pair of Lotka–Volterra competition equations (Otto & Day 2007) with carrying capacity and maximum growth rate normalized to 1 (and the remaining parameters are scaled appropriately). In contrast, a pure cooperative wild-type lineage expends resources on enhancing both its net growth rate and carrying capacity, given *b* > *x* (e.g. through the secretion of shared exoproducts). Despite gains to both growth rate and carrying capacity, a stability analysis (Otto & Day 2007) demonstrates that a population of pure cooperators (at carrying capacity, W* = 1 + *b* −*x*) is vulnerable to invasion by rare cheats, and that the only stable equilibrium in model one is pure cheats, at C* = 1 and extinction of the wild-type, W* = 0 (see also André & Godelle 2005). From a therapeutic perspective, the replacement of *W* by *C* leads to a reduction of bacterial density, and potentially more significantly, the cessation of cooperative virulence factor production (figure 4*a*). This predicted invasion of cheats, with a subsequent reduction of virulence has been observed with QS of cheats in the bacterium *P. aeruginosa* when infecting mice (Rumbaugh *et al*. 2009).

### (b) Trojan horse cheats

The ability of a cheat lineage to invade a patch (i.e. a host) of cooperators opens the potential for hitch-hiking useful traits (e.g. antibiotic vulnerabilities) along with the socially dominant cheat strategy into the microbial pathogen population—a Trojan horse cheat. This is analogous in some ways to the suggestion that selfish genetic elements can be used to genetically engineer natural populations (Turelli & Hoffman 1999; Burt 2003). We modify our above model by inserting an engineered vulnerability into the cheat lineage, imposing a direct growth cost *q* (if the engineered trait provides a direct growth benefit, this would be represented by a negative *q*), yielding.

2.2
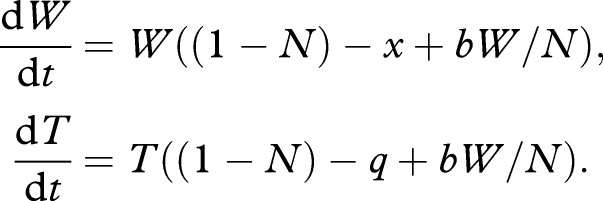


In the case of an engineered susceptibility to a specific antibiotic treatment, it is important to delay use of the antibiotic treatment until the Trojan horse cheat has established within the host, otherwise the costs imposed on the Trojan horse cheat by the antibiotic would overwhelm the gains via social cheating, and the Trojan horse would fail to invade. While any remaining wild-type are likely to enjoy a competitive advantage once the Trojan horse controlling antibiotic is administered, at this point the wild-type is reduced in density following the earlier Trojan horse invasion. This reduction in parasite density could aid any other intervention strategy, emphasizing that such Trojan horse cheats could be useful as part of a larger strategy.

An alternate class of Trojan horse traits could bring more immediate therapeutic advantages to Trojan horse invasion, if the Trojan horse directly sows the seeds of destruction for both the wild-type and itself. For example, a Trojan horse lineage engineered to produce an antimicrobial toxin that kills when at sufficient density (i.e. the toxin is under QS-dependent regulation), both *W* and *T*. In this case, the Trojan horse lineage generates a socially mediated cost, proportionate to its population share (*T/N*), thus when the Trojan horse is initially rare, it can largely escape this social cost, and still invade. Alternatively, a Trojan horse could be engineered to produce an antimicrobial toxin upon addition of a certain chemical. In this case, a toxin gene would be placed under the control of an inducible promoter and only activated when the promoter is activated by the inducer chemical. In this case it would be advantageous to allow the Trojan horse to significantly invade the population before activating its deadly cargo. Weighing the socially mediated cost by *a*, in the case of a constitutive toxin-producer, we have

2.3
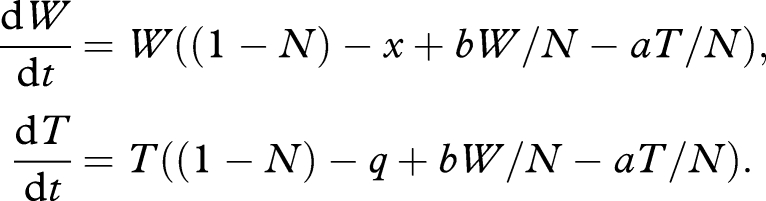


In this non-spatial model, the outcome of competition is entirely defined by the relative magnitudes of the direct effects *x* and *q*. If *x* > *q* (if the cost of wild-type cooperation is greater than the cost of the Trojan horse engineered vulnerability), then the cheat lineage will again invade and dominate within the host (tending to a carrying capacity of T* = 1 − *q* − *a*; figure 4*c*) driving the wild-type to extinction, otherwise the cheat will fail to invade.

The Trojan horse cheat offers potentially multiple therapeutic gains. Given *a* = 0 (equation (2.2)), Trojan horse invasion leads to a reduction of total bacterial density (tending to T* = 1 − *q*, from W* = 1 + *b* − *x*), the loss of virulence factor production associated with wild-type cooperation, and the fixation of an engineered vulnerability (e.g. antibiotic susceptibility) within the bacterial population (figure 4*a*). The generation of a socially mediated cost by the Trojan horse lineage (*a* > 0; equation (2.3)) offers the additional benefit of further depressing the remaining Trojan horse bacterial lineage (figure 4*c*), potentially to extinction (if *a* > 1 − *q*).

### (c) Trojan horse cheat in a spatially structured host

The above model is very favourable to our basic argument, as cheats that produce no or less exoproducts can readily invade an unstructured population of cooperators (e.g. Griffin *et al*. 2004; Brockhurst *et al*. 2007; Diggle *et al*. 2007*b*; Ross-Gillespie *et al*. 2007; Sandoz *et al*. 2007; Kümmerli *et al*. 2009*a*,*b*). However, while unstructured populations occur in shaken laboratory cultures, infections in hosts are likely to be spatially structured (Rumbaugh *et al*. 2009). In order to consider the additional challenge of cheat invasion into a spatially structured host, we assume that within-host interactions are non-random. Specifically, we assume that individual pathogens interact with their kin with probability *r*, and otherwise interact with individuals drawn at random from the entire within-host population (e.g. Ross-Gillespie *et al*. 2007), yielding2.4
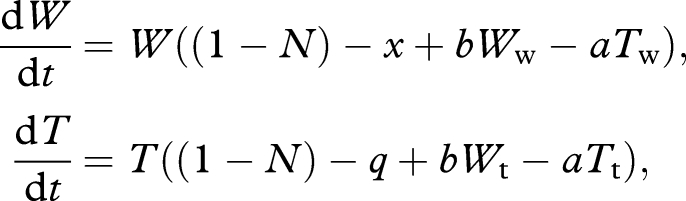
where *W*_w_ and *T*_w_ refer to the average local density of *W* and *T*, in the neighbourhood of a wild-type, and *W*_t_ and *T*_t_ refer to the average local density of *W* and *T*, in the neighbourhood of a Trojan horse. Specifically, we have *W*_w_ = *r* + (1 − *r*) *W/N*; *W*_t_ = (1 − *r*) *W/N*; *T*_w_ = (1 − *r*)*T/N* and *T*_t_ = *r* + (1 − *r*)*T/N*. When *r* = 0, we recover the well-mixed model 1, with *W*_w_ = *W*_t_ = *W/N* and *T*_w_ = *T*_t_ = *T/N*, and when *r* = 1 we have complete separation of the strains, with *W*_w_ = *T*_t_ = 1 and *T*_w_ = *W*_t_ = 0. Note the demographic term (1 − *N*) remains global (unmodified by *r*), reflecting constraints on the remaining ‘host space’. We can interpret *N* in the spatial model as the proportion of distinct potential infection sites within a host that are infected, and *r* as a measure of within-site relatedness (where social interactions take place).

When *a* = *q* = 0, we recover a structured version of equation (2.1), wild-type versus cheats *C*. Given sufficiently low *r*, the sole stable equilibrium remains pure *C* (with W* = 0), however if *rb* > *x* (cf. Hamilton's Rule (Hamilton 1963, 1964)); then pure *W* (at W* = 1 + *b* − *x*) becomes the sole attractor, highlighting a standard result of social evolution theory that spatial structure promotes cooperation (figure 4*b* illustrates an intermediate case, where limited within-host structuring slows invasion of the cheat; reviewed by West *et al*. 2002; Lehmann & Keller 2006). Given that this result runs counter to our goal of developing a therapeutic cheat agent, how can we proceed?

Turning to the full Trojan horse model (equation (2.4)), we see that the condition for wild-type vulnerability to invasion (and for Trojan horse stability) becomes *r*(*b* + *a*) < *x*−*q*, again favoured by low within-host structure *r*. The Trojan horse cheat's invasive ability is further weakened by its direct costs *q*, and also any socially mediated costs *a*, as these costs are now more heavily felt by the invasive Trojan horse lineage (figure 4*d*).

Hamilton's rule also shows how ‘ultra-cooperators’ could theoretically be used to invade a population, although this is unlikely to be a practical option. If the Trojan horse delivers sufficient social *benefits* (i.e. if −*a* > *b*, the Trojan horse is *more* cooperative than the wild-type), then increasing population structure *r* will favour invasion of the Trojan horse lineage. In this scenario, we have a negative *a* (for example the Trojan horse produces a growth-enhancing exoproduct) and the Trojan horse would lead to an *increase* in the within-host microbial density (from W* = 1 + *b* − *x* to T* = 1 − *a* − *q* and W* = 0; figure 4*e*,*f*), but the remaining therapeutic gains would still stand: loss of virulence factor production (extinction of the wild-type strain), fixation of engineered vulnerability (e.g. antibiotic sensitivity). If *a* is negative, the Trojan horse becomes a cooperative lineage and therefore we are exploring a reversal of our original premise, here proposing a cooperator to invade a structured social population, and consequently we find that increasing within-host structure enhances the invisibility of the Trojan horse cooperator (figure 4*e*,*f*). While this demonstrates the theoretical plausibility of using an ultra-cooperator to invade a structured population (of cooperators), we note that there are likely to be practical problems that make this much less useful than the scenario of a cheat invading. In particular, natural selection is likely to have led to the wild-type producing exoproducts at a rate that cannot be invaded (i.e. if greater cooperation was favoured, then lineages would already be doing it), or it may be hard to genetically engineer such cooperators.

### (d) Bacteriocinogen cheat invasion

The above models have considered the invasion of individuals that differ in their rate of production of a cooperative exoproduct. Another possibility is to exploit a different type of social trait—the production of anti-competitor chemicals or bacteriocins (Riley & Gordon 1999; Riley & Wertz 2002). Bacteriocin-producing lineages carry two tightly linked traits. First, they carry genes coding for a small peptide anti-competitor toxin, a bacteriocin. Second, they carry genes conferring immunity to this bacteriocin, ensuring that the toxicity is experienced preferentially by non-kin.

A broad strand of experimental and theoretical work on the ecology of bacteriocin-mediated competition highlights the importance of spatial structure in mediating the outcome of competition, with the consensus recognizing that structured environments promote the invasion of rare killers by increasing the local density of chemical weapons to an effective dose (Chao & Levin 1981; Levin 1988; Frank 1994; Durrett & Levin 1997; Gordon & Riley 1999; Gardner *et al*. 2004). Gardner *et al*. (2004) go on to illustrate that the production of anti-competitor chemical weapons can be understood as an example of microbial spite. A spiteful trait imposes costs on both actor and recipient, and is favoured when it is preferentially directed at non-relatives, because this has the indirect benefit of freeing-up resources for relatives (Hamilton 1970; Lehmann *et al*. 2006; Gardner *et al*. 2007*a*). Bacteriocin production can be considered a spiteful trait as it has a negative fitness impact on the actor cell producing the toxin (suicidal cell lysis to release the toxins), imposes a clear cost on recipient cells that are sensitive to the action of the toxin, while freeing-up resources for resistant relatives (Gardner & West 2004; Gardner *et al*. 2004; Inglis *et al*. 2009). Note that the indirect benefit of freeing-up resources is greatest when populations are structured, with local competition for resources.

We begin with a non-spatial treatment of competition between a bacteriocinogenic cheat and wild-type. Consider a cheat lineage *B* that is also bacteriocinogenic, producing (at a cost *q*) an anti-wild-type compound with efficacy *e* (scaled by density of bacteriocinogen, *B/N*). In a well-mixed host, we have

2.5
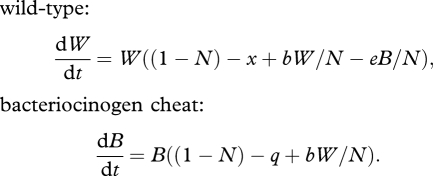


We again assume *b* > *x* (investment in public goods increases carrying capacity) and additionally that *e* > *q* (investment in bacteriocin gives relative advantage when dominant). Note when *x* = *b* = 0 (no public goods interaction), we recover classic bacteriocin-mediated competition models with frequency threshold to invasion at *B/N* = *q/e* (Frank 1994; Durrett & Levin 1997; Brown *et al*. 2006). A stability analysis illustrates that pure wild-type is locally stable (at W* = 1 + *b* − *x*) if *q* > *x* (i.e. if bacteriocin is expensive relative to public good of *W* lineage). Pure cheat *B* is stable (at B* = 1 − *q*) if *x* + *e* > *q* (killing compensates for costs) and *q* < 1 (B* is sustainable). Note that if *x* + *e* > *q* > *x*, then both pure equilibria are stable (bistability, with threshold defined by unstable equilibrium {W*, B*}, where B*/N* = (*q*−*x*)/*e*). Note that this threshold frequency of invaders (*q − x*)/*e* simplifies to the classic threshold frequency of *q/e* when *x* = 0. Increasing *x* (increasing the additional cheat benefit to *B*) decreases the threshold to disappearance when *x* > *q*: i.e. given a sufficient cheating advantage, the rarity threshold to bacteriocin-mediated invasion can be overcome (i.e. the cheating and spiteful traits interact synergistically, see also Brown & Taylor submitted).

**Figure 5.**
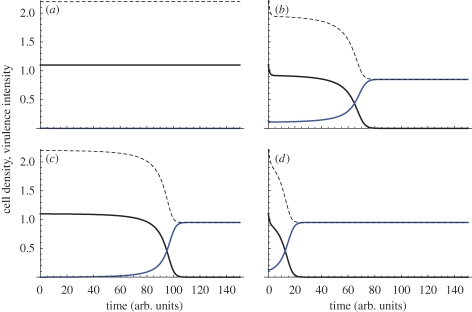
Invasion of bacteriocinogen cheats into an established resident infection and consequent virulence (equation (2.5)). Black lines, wild-type (*W*). Blue lines, bacteriocinogenic (*B*). Dotted lines, virulence (a weighted sum of wild-type and cheat density). (*a*,*b*) Bacteriocinogen has higher direct costs (*x* = 0.1, *q* = 0.15). (*c*,*d*) Bacteriocinogen has lower direct costs, and is an effective social cheat (*x* = 0.1, *q* = 0.05). (*a*,*c*) Bacteriocinogen is initially rare (*W*(0) = 1.1, *B*(0) = 0.001). (*b*,*d*) Bacteriocinogen is initially more common (*W*(0) = 1.1, *B*(0) = 0.13). Virulence = 2*W* + *T*. Other parameters: *b* = 0.2; *e* = 0.5.

Figure 5*a*,*b* illustrates the frequency-dependent fate of bacteriocinogen invasion when the invading *B* lineage pays a significant direct cost, *q* > *x* (e.g. *B* carries a costly Trojan horse trait. A similar and classic result would hold for the case where *B* and *W* are identical with respect to their public goods provision). In this particular illustration, the unstable equilibrium is at B*/*N* = 0.1; below this frequency, invasion fails (figure 5*a*), above this frequency the invader dominates (figure 5*b*). In contrast, figure 5*c*,*d* illustrates the more favourable case, where the direct costs to the bacteriocinogenic lineage are small (*q* < *x*), and consequently the killer lineage can invade from any frequency. Figure 5 illustrates that modifying multiple social traits in conjunction (here, public goods and bacteriocin production) can generate more favourable outcomes (here, invasion from rare due to public goods cheating, and accelerating exclusion of wild-type due bacteriocin production).

Modelling structured within-host (or within-patch) ecological dynamics of bacteriocin-mediated competition is more complex than the previous structured social interactions in §2*c*, and is usually addressed via simulations (Frank 1994; Durrett & Levin 1997; Czárán *et al*. 2002) or a heuristic approach (Gardner *et al*. 2004). Across these models we find consensus with the experimental findings that spatial structuring allows bacteriocinogen invasion from rare (Chao & Levin 1981). The effect of spatial structuring on the invisibility of a cooperative wild-type by a bacteriocinogenic cheat remains an open question. On the one hand, invasibility via bacteriocin production will be enhanced by spatial structuring (see above), while on the other hand, invasibility via social cheating will be reduced by within-host structuring (figure 4*a*,*b*).

It is worth noting that there are other mechanisms of microbial spite, with qualitatively different invasion dynamics (Brown *et al*. 2009), which could be engineered into a Trojan horse therapeutic strain. In particular, a strategy of coupling a Trojan horse with a temperate phages may offer significant advantages for the invasion of unstructured foci of infection (Brown *et al*. 2006). Temperate phages are viruses of bacteria that can be transmitted either vertically or horizontally. Infection of susceptible bacteria by temperate phages can result in two possible outcomes; the most common is the lytic cycle (rapid host lysis and production of numerous horizontally transmissible viral particles). Very rarely, however, the phage can lysogenize the host, persisting in a dormant state while allowing the survival of the infected bacteria. This dormant phage is then replicated with the bacterial genome, and thus vertically transmitted upon bacterial division. Furthermore, this vertically transmitted carried-phage provides immunity to its carrier-bacteria against further horizontal infection by this phage (Adams 1959; Campbell 1996). Upon rare spontaneous induction of the carried-phage, viral progeny are released through host lysis.

Temperate phages have been demonstrated to function analogously to bacteriocins (Bossi *et al*. 2003; Brown *et al*. 2006; Joo *et al*. 2006), by differentially killing susceptible (non-lysogen) bacteria. Brown *et al*. (2006) further demonstrated experimentally that unlike bacteriocinogenic lineages (see above), lysogenic bacteria can invade rapidly from rare into unstructured environments, due to the ability of the released phage to amplify on susceptibles. Here, we consider the fate of a candidate theraputic cheat lineage, that carries an additional temperate phage weapon that is active against the target resident bacteria. We begin with a simple non-spatial treatment tracking the densities of wild-type *W*, lysogen cheat *C* and free viral propagules *V*,

2.6
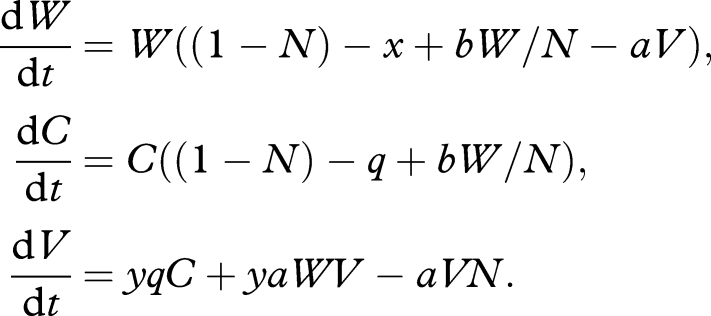


Following Brown *et al*. (2006), we find that pure wild-type is always unstable (so long as burst size *y* is greater than 1), and that pure *C* and *V* are stable if *q* < 1 and *y* > 1, therefore we have a simple general outcome in the non-spatial case of extinction of the wild-type *W*, irrespective of the social behaviour (governed by *x* and *b*) of the resident wild-type. Understanding how microbial competition mediated by temperate phage is modified by within-host spatial structure is an open question, however it is likely that in contrast to the bacteriocin case, spatial structuring will act to dampen invasion by slowing the viral epidemic, due to the separation of susceptibles from free virus.

## 3. Discussion

We have applied social evolution theory to suggest novel intervention strategies in the treatment of bacterial infection: (i) the introduction of an invasive cheat that does not contribute to the production of a virulence factor can lead to a reduction in parasite virulence, as well as a reduced bacterial population size, that may make the infection more susceptible to other intervention strategies (§2*a*, figure 2); (ii) cheats could be used as Trojan horses to introduce useful traits such as antibiotic sensitivity into the population (§2*b*, figure 3); (iii) social dominance by a more benign and controllable microbe could be achieved by harnessing allelopathic traits to the therapeutic strain, that are active against the resident pathogen (e.g. bacteriocins, temperate phages; §2*d*,*e*). These different possibilities may interact synergistically (e.g. a bacteriocin-producing cheat), or with other strategies in a larger intervention plan (see also Brown & Taylor submitted). We have used a few simple examples to illustrate the general points, and there are a range of other social behaviours that could be exploited, including lower levels of persistence (Gardner *et al*. 2007*b*), cell death (Ackermann *et al*. 2008) as well as cheats defective in multiple social traits. Furthermore, this approach could also be useful in other areas such as industry (e.g. problems with biofilms) or agriculture (e.g. treating plant pathogens).

All the different scenarios that we have suggested have advantages and disadvantages, which should be considered for specific cases (table 1). Invasion of an exoproduct cheat is more likely when microbial populations are relatively unstructured within-hosts (§2*c*) where the exoproduct diffuses over larger distances (lower *r*), and when the cost of exoproduct production is high (higher *x*). Note that the degree of within-host structuring can be temporarily reduced to favour cheat invasion through the use of a sufficiently diffuse mechanism of inoculation, for example using an aerosol spray. As the invasion of cheats proceeds, within-host structuring is liable to increase (with cheats and cooperators increasingly segregated in distinct foci), however, by this time a significant theraputic gain in overall virulence factor reduction may have been achieved. A potential complication is that some exoproducts show specificity in their uptake: different strains of *P. aeruginosa* produce different forms of pyoverdine, and strains are best able to take up their form of pyoverdine (Meyer *et al*. 1997; Smith *et al*. 2005). Exoproducts that did not involve such specificity (e.g. proteases) would be more useful, because it would not be necessary to match the ‘type’ of the cheat and the cooperator that they are to invade. Another complication that we have not considered is that the genes for many social traits can be on plasmids that are horizontally transmitted between cells (Smith 2001). For example, one of the most virulent strains of bacteria to be reported recently is the strain of MRSA USA300, in which the gene responsible for antibiotic resistance is located on a horizontally transmissible plasmid. Plasmids may represent another avenue for the Trojan horse approach.

Given that cheats producing less or no exoproducts can invade infections, why are cheats not more common in natural infections? It is important here to distinguish between within-host and between-host (patch) dynamics (Kümmerli *et al*. 2009*a*). Within hosts, cheats can potentially invade (Rumbaugh *et al*. 2009). However, this leads to reduced bacterial growth (Rumbaugh *et al*. 2009) and hence a lower transmission to future hosts—transmission success will depend on the proportion of cooperator cells in an infection (figure 2). The overall balance of these two opposing forces will depend upon the extent to which cheats and cooperators can occur in the host (Brown *et al*. 2002; West & Buckling 2003; West *et al*. 2006). If the population dynamics lead to mixed infections with both types (high strain diversity within hosts), then within-host dynamics will favour cheats. In contrast, if infections tend to be only cheats or cooperators (low strain diversity within hosts), then the between-host dynamics will favour cooperators. These two conditions correspond to relatedness being relatively low or high, respectively, and are another way of conceptualizing the familiar result that altruistic cooperation is favoured by high relatedness (Hamilton 1964). Even when natural population dynamics lead to high relatedness that favours cooperative virulence traits, it is still possible that a cheat could invade. In chronic infections, where there is a relatively low level of transmission between hosts, we predict that cheater strains could invade in natural populations. There is some evidence to support this prediction: biofilm formation is relatively poor in strains from older infections (Lee *et al*. 2005) and mutations occurring post-colonization are found in genes controlling social behaviours, such as public good production and QS (Smith *et al*. 2006). The introduction of cheats into these infections may be less effective in the ways described in this paper, as there may not be a sufficient amount of cooperation going on in the infective population for the introduced cheats to exploit. Another general point is that we assume throughout that the mechanics of host exploitation are inherently cooperative, and therefore, that the introduction of cheats will reduce virulence. For pathogens that do not engage in cooperative virulence mechanisms, social cheats are predicted to be more virulent (Frank 1996) and our theraputic approach would not work.

With any new class of anti-infective therapy, it is essential to consider the potential risk of resistance evolution—see discussions of phage therapy (Levin & Bull 2004) and antimicrobial peptides (Bell & Gouyon 2003; Perron *et al*. 2006). André & Godelle (2005) have also argued that by attacking social traits directly (for example, by disrupting QS regulation), the selective pressure driving the emergence of resistance traits to social perturbations can be dramatically reduced relative to classic antimicrobials, because it is hard for rare cooperators to invade populations of cheaters. Selection will be unlikely to favour the restoration of cooperative function in individuals that have evolved resistance to cheats. While the same argument holds for our particular brand of social perturbation, the emergence of resistance is still conceivable. Natural anti-cheat resistance traits are widespread, for example mechanisms of specificity (discussed above) effectively remove cheats from the pool of shared goodies produced by a focal cooperative lineage. For lineages without inbuilt mechanisms of cheater resistance (most likely reflecting a transmission ecology characterized by high bottlenecking and consequently a dominance of single-genotype infections), we anticipate the greatest initial success for our strategy, and also the greatest long-term risk of resistance evolution.

Another point of departure from classic chemical mechanisms of microbial control concerns the within-host dynamics of our control agent. In this regard, our proposed cheat therapy most closely resembles phage therapy, where a live natural enemy is administered to control an infection (rather than a live social parasite). In both cases, the control agent is able, in principle, to replicate at the site of interest, within infection, therefore offering significant gains over a chemical agent that must be introduced en masse, often at damagingly high doses, at a remote site of entry. However, the slow development of phage therapy over many decades offers further notes of caution (Levin & Bull 2004): replication of the control agent may only be possible under certain physiological states of both resident and control agent, and the ability of the control agent to reach infection sites (the ‘pharmacokinetics’ of social parasites) may be severely limited, relative to classic chemical control agents (although see Rumbaugh *et al*. 2009). A further cause for advantage—and cause for concern—shared by phage and social parasite therapy is the potential ability of the control agent to co-evolve with its target, potentially prolonging efficacy, but also raising the spectre of unintended consequences.
